# Impact of an additional chronic BDNF reduction on learning performance in an Alzheimer mouse model

**DOI:** 10.3389/fnbeh.2015.00058

**Published:** 2015-03-20

**Authors:** Laura Psotta, Carolin Rockahr, Michael Gruss, Elmar Kirches, Katharina Braun, Volkmar Lessmann, Jörg Bock, Thomas Endres

**Affiliations:** ^1^Institute of Physiology, Medical Faculty, Otto-von-Guericke-University MagdeburgMagdeburg, Germany; ^2^Department of Zoology/Developmental Neurobiology, Institute of Biology, Faculty of Natural Sciences, Otto-von-Guericke University MagdeburgMagdeburg, Germany; ^3^Institute of Neuropathology, Faculty of Medicine, Otto-von-Guericke University MagdeburgMagdeburg, Germany; ^4^Center for Behavioral Brain Sciences (CBBS), Otto-von-Guericke University MagdeburgMagdeburg, Germany

**Keywords:** BDNF, Alzheimer’s disease, active avoidance, water maze, object recognition, APP/PS1

## Abstract

There is increasing evidence that brain-derived neurotrophic factor (BDNF) plays a crucial role in Alzheimer’s disease (AD) pathology. A number of studies demonstrated that AD patients exhibit reduced BDNF levels in the brain and the blood serum, and in addition, several animal-based studies indicated a potential protective effect of BDNF against Aβ-induced neurotoxicity. In order to further investigate the role of BDNF in the etiology of AD, we created a novel mouse model by crossing a well-established AD mouse model (APP/PS1) with a mouse exhibiting a chronic BDNF deficiency (BDNF^+/−^). This new triple transgenic mouse model enabled us to further analyze the role of BDNF in AD *in vivo*. We reasoned that in case BDNF has a protective effect against AD pathology, an AD-like phenotype in our new mouse model should occur earlier and/or in more severity than in the APP/PS1-mice. Indeed, the behavioral analysis revealed that the APP/PS1-BDNF^+/−^-mice show an earlier onset of learning impairments in a two-way active avoidance task in comparison to APP/PS1- and BDNF^+/−^-mice. However in the Morris water maze (MWM) test, we could not observe an overall aggrevated impairment in spatial learning and also short-term memory in an object recognition task remained intact in all tested mouse lines. In addition to the behavioral experiments, we analyzed the amyloid plaque pathology in the APP/PS1 and APP/PS1-BDNF^+/−^-mice and observed a comparable plaque density in the two genotypes. Moreover, our results revealed a higher plaque density in prefrontal cortical compared to hippocampal brain regions. Our data reveal that higher cognitive tasks requiring the recruitment of cortical networks appear to be more severely affected in our new mouse model than learning tasks requiring mainly sub-cortical networks. Furthermore, our observations of an accelerated impairment in active avoidance learning in APP/PS1-BDNF^+/−^-mice further supports the hypothesis that BDNF deficiency amplifies AD-related cognitive dysfunctions.

## Introduction

Alzheimer’s disease (AD) is the most common form of dementia (Kawas and Corrada, 2006), resulting in a progressive decline of cognitive functions (Blennow et al., 2006). While the cellular and molecular mechanisms that underlie AD are still not well understood, some neuropathological hallmarks were already identified. These include neurofibrillary tangles consisting of aggregated hyperphosphorylated tau protein and neuritic plaques characterized by an overload of mainly insoluble Aβ peptides (e.g., Hardy and Higgins, 1992). Mutations in the amyloid precursor protein (APP) and the presenilin genes (PS1 and PS2), which are both linked to the familiar form of AD (e.g., Goate et al., 1991), have been described to lead to a massive increase in cytotoxic Aβ_40_ and Aβ_42_ oligomers (e.g., Walker et al., 2005; Theuns et al., 2006).

One of the main objectives of Alzheimer research is to identify novel therapeutic approaches alleviating cognitive deficits. In this context, focusing on neurotrophin signaling appears to be a promising approach. Brain-derived neurotrophic factor (BDNF) is known to play a critical role in the survival, differentiation and synaptic plasticity of mammalian neurons (see e.g., Cowansage et al., 2010; Edelmann et al., 2014). The cellular actions of BDNF are mediated mainly through TrkB-receptors (summarized e.g., in Park and Poo, 2013). In the adult mammalian brain BDNF is most prominently expressed in the entorhinal cortex and in the hippocampus (Phillips et al., 1990; Ferrer et al., 1999; Quartu et al., 1999), brain areas that are also affected by neuronal loss in AD (Peng et al., 2005). Furthermore, AD patients have been shown to exhibit decreased BDNF levels in these regions (Phillips et al., 1991; Murray et al., 1994; Amoureux et al., 1997), which is mirrored by reduced blood serum BDNF levels in AD patients (Laske et al., 2006). *In vitro* experiments demonstrated that BDNF exerts several neuroprotective effects by reducing the cytotoxic effects of Aβ_42_ (Arancibia et al., 2008) and by stimulating the non-amyloidogenic pathway, resulting in a reduction of toxic Aβ species (Scheuner et al., 1996; Fu et al., 2002; Nishitomi et al., 2006; Thornton et al., 2006; Rohe et al., 2009). In rodent and primate models of AD it has been shown that acute application of BDNF protein can partially rescue Aβ_42_-induced neurodegenerative changes (Nagahara et al., 2009). Furthermore, studies in AD mouse models revealed that activation of the TrkB-receptor via application of the recently discovered TrkB-receptor agonist 7,8-Dihydroxyflavone rescued learning and memory deficits and reduced Aβ levels in the brain (Devi and Ohno, 2012; Castello et al., 2014; Zhang et al., 2014).

Taken together, several lines of evidence suggest that direct application of BDNF or activation of TrkB-receptors exert a protective effect against AD pathology and that the expression of BDNF seems to be down-regulated in AD patients. However, the mechanisms explaining how changes in BDNF protein levels might contribute to the occurrence and severity of AD pathology are still unknown. To further investigate how altered BDNF levels might contribute to the progression of AD pathology, we created a novel mouse model that combines AD pathology with chronically reduced BDNF levels. We crossed an APP/PS1 mouse model (Radde et al., 2006) with a heterozygous BDNF knock out (BDNF^+/−^) mouse (Korte et al., 1995), in which BDNF protein levels are reduced to roughly 50% of the BDNF levels observed in wildtype animals (see e.g., Psotta et al., 2013). We hypothesized that, if endogenous BDNF has a protective impact against AD pathology, AD-like symptoms should occur in more severity and/or occur earlier in live history in this novel mouse model compared to the classical APP/PS1-mouse line. Therefore, we tested adult (5 months) and aged (12 months) animals for their cognitive abilities in the novel object recognition (NOR) task, the two-way active avoidance paradigm and the Morris water maze (MWM). Also, open field (OF) and elevated plus maze (EPM) tests were conducted, to test for possible differences in explorative activity and anxiety levels. For a more detailed analyis of the neuropathological characteristics in the investigated mouse lines, amyloid plaque load in the hippocampus and prefrontal cortex were analyzed by quantitative immunhistochemistry.

## Material and Methods

### Animals

Male mice of four different genotypes at the age of 5 and 12 months were used: APP/PS1-mice carrying the human APP gene locus with the Swedish double mutation KM670/671NL and L166P mutated human PS1 under control of the Thy1 minigene promoter (Radde et al., 2006); BDNF^+/−^-mice (Korte et al., 1995); APP/PS1-BDNF^+/−^-mice: crossbreeding between APP/PS1- and BDNF^+/−^-mice. All mouse lines were bred on C57BL/6J genetic background (Charles River, Sulzfeld, Germany). Mice were kept under standard conditions with 2–4 animals per cage in a dark-light cycle of 12 h (light on at 7:00 a.m.) with food and water *ad libitum*. The offsprings of APP/PS1 × BDNF^+/−^ breeding pairs were weaned between 3–4 weeks of age and maintained in groups of 3–4 animals each. Tail cuts were used for genotyping, according to previously published polymerase chain reaction (PCR) protocols for BDNF^+/−^ (Abidin et al., 2008) and APP/PS1-mice (Radde et al., 2006). All experiments were carried out during the light phase of the animals. The experiments were in accordance with the guidelines for the use of animals in experiments and were approved by the local animal welfare committee (Landesverwaltungsamt Sachsen-Anhalt, 42502-2-1047UniMD and 42502-2-1132UniMD).

### Behavioral Testing

#### Open Field (OF)

Exploratory behavior was tested in an OF arena (50 × 50 × 35 cm^3^). At the beginning of the test individual animals were placed in the center of the arena were allowed to freely explore the maze for five minutes. Exploratory behavior (total distance traveled, time in center) was automatically recorded and analyzed using a video tracking software (AnyMaze, Stoelting Co., Wood Dale, IL). For the analysis of exploratory behavior a virtual center area of 30 × 30 cm^2^ was defined. The center of the animal body was defined as tracking point. After the test, animals were returned to the home cage. The arena was intensively cleaned after each animal to prevent odor cues.

#### Elevated Plus Maze (EPM)

To analyze anxiety-like behavior, an EPM test was performed. The EPM consisted of two open (5 × 30 cm^2^) and two closed (5 × 30 × 15 cm^3^) arms elevated 40 cm from the floor. At the beginning of the experiment animals were placed in the center of the EPM (every animal in the same orientation) and were allowed to freely explore the maze for five minutes. The center of the animal body was defined as tracking point. After each trial the maze was cleaned carefully to prevent odor cues. The parameters time on the open arms and total distance traveled were automatically recorded and analyzed (AnyMaze, Stoelting Co., Wood Dale, IL).

#### Novel Object Recognition (NOR)

To evaluate short-term object memory abilities, a NOR task was performed in a rectangular arena (30 × 45 cm^2^). During the first exposure two identical objects were presented to each individual animal, which was allowed to explore the objects for 10 min. The objects and their position in the arena was pseudorandomized between the different animals. After a 30 min break one of these objects was replaced by a novel object, again the animals were allowed to explore the objects for 10 min. An observer blind to the genotype of the respective animal manually recorded the time the animals explored each object.

#### Morris Water Maze (MWM)

Spatial learning performance was tested in the MWM. The MWM consisted of a circular pool with a diameter of 150 cm filled with opaque water (22 ± 1°C) to hide the escape platform. The platform was placed in the center of one of four quadrants (target quadrant), which virtually divided the maze. White curtains with different patterns surrounded the maze and served as distal visual cues to allow spatial orientation. Experiments were automatically recorded and analyzed by a video tracking software (AnyMaze, Stoelting Co., Wood Dale, IL).

The animals were exposed to four training trials (60 s with an inter-trial-interval of 20 min) per day on four consecutive days. Each individual was placed in the maze at pseudo-randomized starting positions facing the wall of the tank. The time the animals needed to reach the platform (latency) was recorded. 24 h after the last training trial a probe trial was conducted (60 s), in which the platform was removed and memory performance was tested by the time the animals spent in the former target quadrant.

#### Two-way Active Avoidance Training (TWAA)

For testing active avoidance behavior an automated shuttle-box system (TSE Systems, Bad Homburg, Germany) was used. The apparatus was divided into two compartments (14 × 15.5 × 16 cm^3^), which were separated by a non-transparent white Plexiglas wall with an opening to allow access to the other compartment. Training was conducted on five consecutive days and started every day with a habituation phase of three minutes, during which the animals were allowed to freely explore the shuttle box, followed by 50 trials of CS-UCS pairings. A trial started with the presentation of a conditioned stimulus (CS; 4.0 kHz tone, 80 dB SPL; max. 5 s), which was immediately followed by simultaneous presentation of the CS and an unconditioned stimulus (0.3 mA foot-shock, max. 15 s) and an inter-trial interval of 20 s. Successful learning was assessed by counting the number of avoidance responses, which were recorded automatically. An avoidance response was recorded when the mouse moved to the opposite compartment during the initial CS presentation (i.e., within 5 s), but prior to US onset.

For the described behavioral experiments animals were assigned to one of two behavioral test batteries: (1) OF, followed by EPM and after a break of 2 days followed by two-way active avoidance training (TWAA) (*n* = 8–10 per genotype and age group). (2) NOR and after a break of 2 days followed by MWM (*n* = 8–12 per genotype and age group).

### Immunohistochemistry

To gain more detailed information about neuropathological characteristics related to AD we analyzed the amyloid plaque load in all for genotypes (wildtype, APP/PS1, BDNF^+/−^, APP/PS1-BDNF^+/−^) by quantitative immunohistochemistry. For this analysis untrained males at the age of 5 and 12 months were used.

Animals were anesthetized with an intramuscular injection of a ketamine/xylazine mixture (4 + 1), and perfused with tyrode buffer followed by 4% paraformaldehyde as fixative. After perfusion brains were removed and postfixed over night in 4% paraformaldehyde. Using a vibrating blade microtome (vibratome, Leica Biosystems, Wetzlar, Germany) brains were cut into 40 µm slices, washed with phosphate buffered saline (PBS) and incubated with Goat Serum for 1 h, followed by incubation with a monoclonal Beta-Amyloid Antibody (Covance, 1:2000) over night. On the following day, brain slices were washed, incubated with biotinylated goat anti-mouse IgG (Millipore, 1:200) for 1 h followed by incubation with Streptavidin-Biotinylated Horseradish Peroxidase Complex (GE Healthcare, 1:100) for 1 h. Slices were stained with the 3,3′-diaminobenzidine (DAB) method and sections were dehydrated, made transparent and mounted by using conventional methods.

Analysis of amyloid plaque load (plaque density) in the hippocampus and prefrontal cortex was done by using a computerized system (Neurolucida, Micro Bright Field, USA) for the analysis and quantification of microscopic images (Olympus BX51).

### Data Analysis

Statistical analysis was performed using SPSS (version 22.0; SPSS Inc., Chicago, USA). For the OF and EPM a one-way ANOVA with genotype as the main factor was applied, followed by a least significant differences (LSD) *post hoc* test. For the NOR a two-way ANOVA with the main factors object and genotype was performed, followed by a LSD *post hoc* test. For MWM and TWAA a two-way repeated measures ANOVA with main factors day of training and genotype, followed by a LSD *post hoc* test was applied. Wildtype mice were defined as control group for all *post hoc* tests. For the analysis of amyloid plaque density a three-way ANOVA with the main factors age, brain region and genotype was performed. The level of significance was set at *p* < 0.05.

## Results

### Aged but not Adult APP/PS1-mice Show Reduced Exploratory Behavior in the Open Field

**Figure 1:**
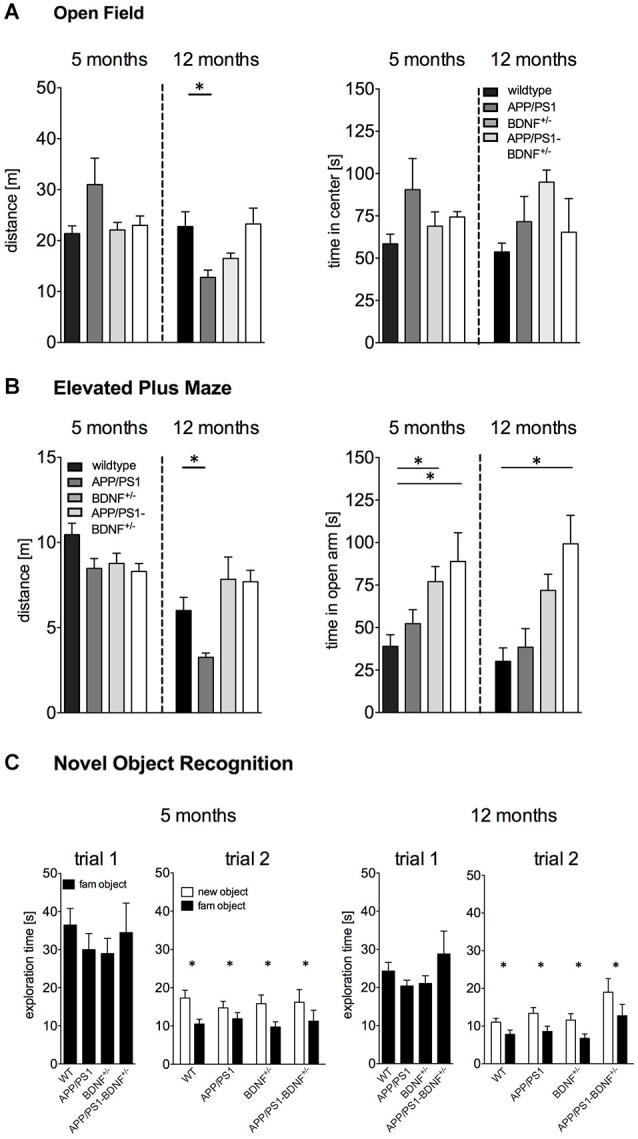
**(A)** Activity (distance moved) and anxiety behavior (time spent in center region) tested in the Open Field of 5 and 12 months old animals. **(B)** Anxiety behavior (time spent in the open arm) in the EPM experiment of 5 and 12 months old animals. **(C)** Short-term memory tested in the Novel Object Recognition test of 5 and 12 months old animals. In all diagrams the mean values + SEM for the four genotypes (WT-, APP/PS1-, BDNF^+/−^- and APP/PS1-BDNF^+/−^-mice) are shown.

Exploratory behavior was assessed by an open OF test (Figure 1A). One-way ANOVA revealed no significant differences in the total distance traveled between the four genotypes (*F*_3,40_ = 2.3, *p* = 0.09) in five months old animals.

In contrast, for twelve months old animals a significant difference between the four genotypes could be observed (*F*_3,37_ = 4.5, *p* = 0.01). A LSD *post hoc* test revealed reduced explorative activity in 12 months old APP/PS1-mice compared to wildtype mice. No significant differences were found for the BDNF^+/−^-mice and the APP/PS1-BDNF^+/−^-mice compared to wildtype mice.

For the time animals spent in the center of the OF one-way ANOVA revealed no significant differences between the four genotypes, neither for 5 nor for 12 months old animals (*F*’s ≤ 2.1; *p*’s ≥ 0.1, Figure 1A).

### Adult and Aged BDNF^+/−^- and APP/PS1-BDNF^+/−^-mice Display Reduced Anxiety Levels in the Elevated Plus Maze

Basal anxiety levels were analyzed by an EPM test (Figure 1B). In five months old animals one-way ANOVA revealed a significant difference between the four genotypes for the time spent on the open arm (*F*_3,34_ = 4.3, *p* = 0.01). LSD *post hoc* test revealed that BDNF^+/−^-mice as well as APP/PS1-BDNF^+/−^-mice spent significantly more time on the open arms compared to wildtype mice.

Similarly, a significant difference in anxiety behavior between the four genotypes was also observed in twelve months old animals (*F*_3,38_ = 7.1, *p* = 0.001). LSD *post hoc* test revealed that APP/PS1-BDNF^+/−^-mice spent significantly more time on the open arm compared to wildtype mice.

For explorative behavior (total distance traveled) during the EPM test one-way ANOVA revealed no significant differences between the genotypes in five months old animals (*F* = 2.7, *p* = 0.07). However, a significant effect for the factor genotype was observed in twelve months old animals (*F*_3,38_ = 5.7, *p* = 0.003). A LSD *post hoc* test revealed lower exploratory behavior in APP/PS1-mice compared to the wildtype but no differences for the BDNF^+/−^-mice and the APP/PS1-BDNF^+/−^-mice compared to wildtype mice.

### No Impairment in Short-Term Memory of BDNF^+/−^-, APP/PS1- and APP/PS1/BDNF^+/−^-mice

In order to analyze the short-term memory abilities of the tested animals, we applied a NOR task (Figure 1C). During the object habituation trial there was no difference in the total exploration time between the four genotypes at any tested age (*F*’s ≤ 0.6, *p*’s ≥ 0.6), indicating that the mutations had no impact on object exploration behavior. After an interval of 30 min one object was replaced by a novel object. All genotypes at both tested ages spent significantly more time exploring the novel object than the familiar object (*F*’s ≥ 11.6, *p*’s < 0.005) indicating an intact short-term memory.

### Old APP/PS1- and APP/PS1-BDNF^+/−^-mice Show Impaired Acquisition in a Spatial Learning Task

**Figure 2:**
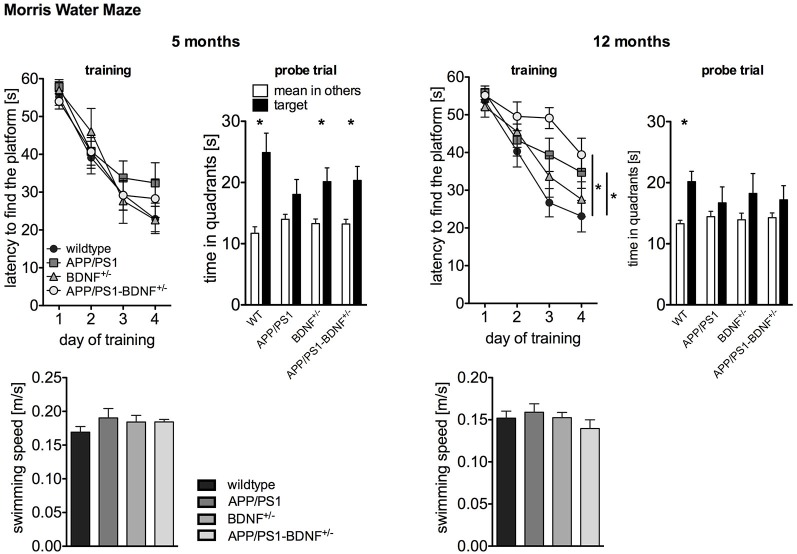
**Spatial learning performance tested by Morris Water Maze of 5 and 12 months old animals**. The left panel depicts the latency to find the platform during the training phase. The right panel shows the time the animals spent in the former target vs. the mean time in the others quadrants during the probe trial performed 24 h after the last training trial. To rule out that the observed differences in latency are due to swimming impairments, we analyzed also the swimming speed of the animals. In all diagrams the mean values (± SEM) of all four genotypes (WT-, APP/PS1-, BDNF^+/−^- and APP/PS1-BDNF^+/−^-mice) are shown.

The spatial learning capacities of the mice were assessed by using the classical MWM paradigm (Figure 2).

As the activity results of the OF and EPM suggested a reduced explorative activity in 12 months old APP/PS1-mice, we analyzed the swimming speed of the animals in order to rule out motor impairments that might interfere with latency measurements (Figure 2). ANOVAs revealed no differences between the tested genotypes at any tested age (*F*’s ≤ 0.9, *p*’s ≥ 0.5), suggesting no swimming impairments in all transgenic animals at any tested age. For five months old animals a two-way repeated measure ANOVA revealed a significant effect of the factor day, indicating a reduced latency to find the platform during the training phase in all four genotypes (day: *F*_3,111_ = 65.5, *p* < 0.001). In contrast, there was neither a significant effect of the factor genotype nor an interaction between the two factors (genotype: *F*_3,37_ = 0.5, *p* = 0.71, day × genotype: *F*_9,111_ = 0.8; *p* = 0.63). This indicates that at this age all genotypes showed a similar learning performance. The analysis of the probe trial revealed a significant effect of the factor quadrant, since the animals spent significantly more time in the target quadrant than in the mean of the other quadrants (quadrant: *F*_1,74_ = 31.1, *p* < 0.001). However, there was no effect of genotype and no interaction of these two factors for the probe trial (genotype: *F*_3,74_ = 0.6, *p* = 0.64; quadrant × genotype: *F*_3,74_ = 2.3, *p* = 0.09). For a more detailed analysis of learning performance during the probe trial the preference for the target quadrant was tested in each genotype with *t*-tests. This approach revealed a reliable memory of the former platform position for wildtype, BDNF^+/−^- and APP/PS1-BDNF^+/−^-mice, since these lines displayed a significant preference for the target quadrant but not for the APP/PS1-mice.

At twelve months of age a two-way repeated measures ANOVA revealed significant effects for the factors day and genotype as well as a significant interaction between these two factors (day: *F*_3,120_ = 45.7, *p* < 0.001; genotype: *F*_3,40_ = 3.4, *p* = 0.03; day × genotype: *F*_9,120_ = 2.0, *p* = 0.04). This indicates a different learning performance over the training days dependent on the genotype. LSD *post hoc* analysis revealed that compared to wildtype mice APP/PS1- as well as APP/PS1-BDNF^+/−^-mice showed significantly longer latencies to reach the platform. The analysis of the probe trial, revealed a significant effect for the factor quadrant, but neither a significant effect for the factor genotype nor an interaction of the two factors (quadrant: *F*_1,80_ = 9.99, *p* = 0.002; genotype: *F*_3,80_ = 0.2, *p* = 0.91; quadrant × genotype: *F*_3,80_ = 0.7, *p* = 0.56). Again, for a more detailed analysis of learning performance during the probe trial the preference for the target quadrant was tested in each genotype with *t*-tests. This revealed a reliable memory of the former platform position only in wildtype mice, since only these mice displayed a significant preference for the target quadrant.

### Early Onset of Impaired Active Avoidance Learning in APP/PS1-BDNF^+/−^-mice

**Figure 3:**
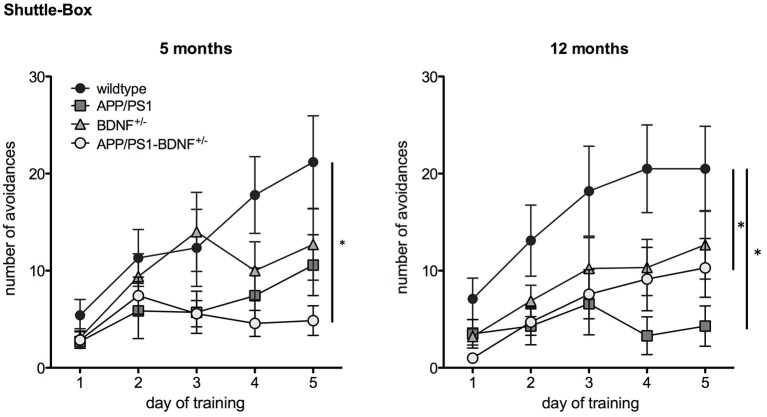
**Learning performance in a Two Way Active Avoidance task (Shuttle Box) in 5 and 12 months old animals**. Diagrams show mean values (± SEM) of the number of avoidance reactions (correct responses) over the five training days of all four genotypes (WT-, APP/PS1-, BDNF^+/−^- and APP/PS1-BDNF^+/−^-mice).

A TWAA test (Figure 3) was employed to investigate negative feedback learning. At five months of age a two-way repeated measures ANOVA revealed a significant effect of the factor day, indicating an increase in learning performance over the five training days for all genotypes (*F*_4,120_ = 12.7, *p* < 0.001). A trend for the factor genotype (*F*_3,30_ = 2.6, *p* = 0.068) and a significant interaction between the factors day and genotype was found (*F*_12,120_ = 2.4, *p* = 0.03), indicating differences in avoidance learning between the four genotypes over the training period. A LSD *post hoc* test revealed that only APP/PS1-BDNF^+/−^-mice showed significantly less avoidance responses compared to wildtype mice indicating impaired ability to learn this particular task and being in accordance with the hypothesis that additional reduction of BDNF can aggravate the memory impairment of APP/PS1-mice.

In twelve months old animals two-way repeated measures ANOVA revealed a significant effect for the factor day, indicating an increase in learning performance over the five training days ( *F*_4,144_ = 16.4, *p* < 0.001). Additionally, a significant effect was found for the factor genotype (genotype: *F*_3,36_ = 4.1, *p* = 0.01), indicating different learning performance between the four genotypes. No significant effect was found for an interaction between the factors day and genotype (day × genotype: *F*_12,144_ = 2.1, *p* = 0.06). LSD *post hoc* test revealed that APP/PS1- as well as APP/PS1-BDNF^+/−^-mice showed significantly fewer avoidance responses compared to wildtype mice indicating that at this age a reduced learning performance of APP/PS1-mice is already detectable in the absence of an additional reduction of BDNF.

### APP/PS1- as well as APP/PS1-BDNF^+/−^-mice show Higher Amyloid Plaque Density in the mPFC than in the Hippocampus

**Figure 4:**
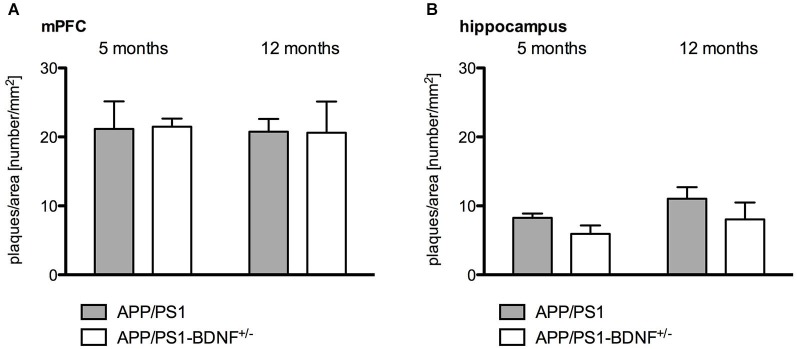
**Quantitative analysis of amyloid plaque density in the mPFC (A) and hippocampus (B) in 5 and 12 months old animals**. Diagrams show mean values (+ SEM) of amyloid plaque desnityfor the two AD genotypes (APP/PS1- and APP/PS1-BDNF^+/−^-mice).

Quantitative immunohistochemistry (Figure 4) was performed to estimate the amyloid plaque load in the mPFC and hippocampus of the analyzed genotypes. At first it became apparent that no amyloid plaques could be detected in wildtype and BDNF^+/−^-mice. Therefore, quantitative comparisons were only performed between APP/PS1- and APP/PS1-BDNF^+/−^-mice. Three-way ANOVA revealded a significant effect only for the factor brain region, (*F*_1,40_ = 99.772, *p* < 0.001), indicating a higher amyloid plaque density in the mPFC compared to the hippocampus. We found no effect for the factors age and genotype and no interactions, which indicates that there is no age-dependent increase of amyloid plaque densities and also a comparable amyloid plaque load in the APP/PS1- and APP/PS1-BDNF^+/−^-mice.

## Discussion

Since there is evidence for an association between altered BDNF signaling and the occurrence and/or intensity of AD pathology, the aim of the present study was to combine these two factors in a novel animal model to study the impact of chronically reduced endogenous BDNF levels on the development of AD pathology *in vivo*. By using this novel mouse model we could show, that the reduction of endogenous BDNF within the APP/PS1-mouse model causes an accelerated onset of learning impairments in a two-way active avoidance task but not in spatial learning in the MWM. Also short term memory for object recognition remained unaffected in APP/PS1-BDNF^+/−^-mice. Quantitative analysis of plaque pathology in the two AD mouse lines revealed no differences between the APP/PS1- and APP/PS1-BDNF^+/−^-mice and also no age-dependent increase between 5 and 12 months of age. However, for both AD mouse lines we found that amyloid plaque density was significantly higher in the mPFC compared to the hippocampus.

Our working hypothesis claimed that BDNF deficiency should accelerate the onset of cognitive impairment and amplify the cognitive deficits displayed by the APP/PS1-mice. As predicted, APP/PS1-BDNF^+/−^-mice showed an earlier onset of impairments in active avoidance learning compared to APP/PS1- and BDNF^+/−^ -mice. At five months of age, avoidance learning was only impaired in APP/PS1-BDNF^+/−^-mice and not yet detectable in APP/PS1- or BDNF^+/−^ -mice, whereas at twelve months of age APP/PS1- and APP/PS1-BDNF^+/−^-mice displayed significant learning deficits compared to wildtype animals. Previous studies testing APP/PS1-mice reported similar age-dependent deficits in avoidance learning in 9 months old mice and in eye blink conditioning at the age of twelve months, which were not yet detectable in three month old animals (Gruart et al., 2008; Lok et al., 2013). Interestingly, no study has tested BDNF^+/−^-mice in the TWAA paradigm so far, even though this task is known to induce BDNF protein expression in several brain areas (Ulloor and Datta, 2005). Even though 12 months old BDNF^+/−^-mice did not perform significantly worse than their wildtype littermates, our data still show a strong trend to an impaired learning performance in these animals at this age. This observation of a probable age-dependent learning decline would be in line with other studies reporting age-dependent learning deficits for BDNF^+/−^-mice in different learning paradigms. For example, a deficit in fear extinction learning was observed in 7 but not 2 months old BDNF^+/−^-mice (Psotta et al., 2013) and BDNF^+/−^-mice exhibit a deficit in amygdala-dependent fear learning, when the animals are older than 3 months of age (Endres and Lessmann, 2012). Together these findings suggest that different learning paradigms, depending on distinct brain areas, all suffer from BDNF deficiency only at later ages.

Importantly, the observed age-dependent decline in the BDNF^+/−^-mice seems to appear later and to a weaker extent than in the APP/PS1- or APP/PS1-BDNF^+/−^-mice, suggesting that the observed accelerated onset of impaired TWAA learning in the young triple transgenic animals is rather due to an accelerated AD-like pathology in combination with chronically reduced BDNF protein levels than simply due to the chronic BDNF reduction alone.

Importantly, for the investigated basal behavioral parameters we didn’t observe any changes of explorative activity and no obvious motoric deficits in the APP/PS1-BDNF^+/−^-mice. In contrast, our EPM data indicate a low anxiety phenotype in the triple mutants. However, it has to be pointed out that this finding was specific for the EPM, no signs of enhanced anxiety-like behavior could be observed in the OF. Taken together, it appears unlikely that the observed learning deficits are due to alterations in basal explorative activity of motor deficts but we cannot completely rule out that the reduced anxiety levels may contribute to the observed learning impairments in the TWAA paradigm. However, this appears unlikely since other studies have shown that low anxiety rats as defined by an enhanced number of entries into the open arms of an EPM performed better in a two-way active avoidance task than high anxiety rats, that showed reduced entries into the open arms of an EPM (Ho et al., 2002).

In contrast to the findings in our new triple mutant mouse model, we found reduced locomotor activity in the 12 months old APP/PS1-mice in the OF as well as in the EPM tests. According to these results one could argue that the BDNF deletion in APP/PS1 mice restored to normal levels of locomotor or exploratory behavior in the APP/PS1-BDNF^+/−^-mice.

However, in the NOR task we didn’t observe any signs of reduced motor activity and explorative behavior in the APP/PS1-mice compared to the other tested animals. Also, exploratory and motor behavior appeared to be normal during MWM, since swimming speed wasn’t reduced in APP/PS1-mice and the latency to find the platform was comparable to the wildtype animals throughout the whole training trials, especially during the first trials that reflect more general locomotor than learning abilities (Figure 3). Thus, our findings, which are supported by previous observations (Radde et al., 2006; Lo et al., 2013a), suggest that there is no general deficit in motor and explorative behavior in APP/PS1-mice.

Also in the MWM paradigm, we observed age-dependent learning deficits. However, in contrast to TWAA learning these learning deficits occurred to a different extent in all tested transgenic animals. While training performance (acquisition) was fully intact in 5 months old animals, the APP/PS1- as well as the APP/PS1-BDNF^+/−^-mice showed significantly reduced training performances at the age of 12 months, thereby the APP/PS1-BDNF^+/−^-mice displayed the worst performance since they showed the highest latencies during the training phase at that age.

For the spatial memory tested in the probe trial in 5 months old animals a reduced learning performance was observed in APP/PS1-mice but not in the other genotypes. However, this finding in the APP/PS1-mice does not reflect a completely impaired memory, since these animals still exhibited a strong tendency towards spending more time in the previous target quadrant (*p* = 0.08). At 12 months of age all tested mutant mouse lines, the BDNF^+/−^-mice, the APP/PS1-mice as well as the triple mutant APP/PS1-BDNF^+/−^ showed an impaired performance in the probe trial when compared to the wildtype animals. But again, there was no complete memory blockade since all animals still showed a trend towards spending more time in the previous target quadrant.

In general, these findings are in line with other studies demonstrating age-dependent spatial learning deficits in APP/PS1-mice occurring around 8/9 months (Radde et al., 2006; Lo et al., 2013a,b), as well as in BDNF^+/−^-mice, occurring around 10–12 months (Linnarsson et al., 1997; Rantamäki et al., 2013). Interestingly, another study using a similar mouse model reported an aggravated AD pathology in the MWM by chronic BDNF reduction (Rantamäki et al., 2013). However, a direct comparison between the two mouse models is difficult since we used a different AD mouse model harboring different APP and PS1 mutations under control of a different promoter, resulting probably in different expression patterns of Aβ and subsequent AD-like pathology.

The quantification of amyloid plaque density in the two AD mouse models revealed no differences between the APP/PS1-mouse and our newly created APP/PS1-BDNF^+/−^-mouse and also no age-dependent differences (5 vs. 12 months). This finding indicates that the amyloid plaque pathology in our new mouse model was not directly affected by the reduced BDNF level, a result that is in line with findings described in the study of Rantamäki et al. (2013).

However, our findings revealed for both analyzed AD mouse models that the amyloid plaque load was higher in the mPFC compared to the hippocampus. These results are comparable to findings of previous studies showing that the classical APP/PS1-mice exhibits a higher (approx. three fold) plaque pathology in the cortex compared to the hippocampus (Radde et al., 2006; Scholtzova et al., 2008; Gengler et al., 2010). In addition it was shown that the onset of plaque pathology occurs later in the hippocampus than in the cortex (Radde et al., 2006). These regional differences in amyloid pathology might explain our finding of an aggravated learning deficit in the TWAA task but not in spatial learning, since these different learning tasks rely on the recruitment of different neuronal networks. Whereas the spatial MWM learning relies mainly on the basal ganglia and hippocampal function (Morris et al., 1982; Cho et al., 1999; Devan and White, 1999; Barry and Commins, 2011), the more complex TWAA learning requires besides several limbic structures (Cain and LeDoux, 2008; Choi et al., 2010) also network activity of higher cortical areas (Martinez et al., 2013; Moscarello and LeDoux, 2013).

Finally, major dysfunctions for short-term memory abilities were neither observed in our novel triple mutant, nor in the two respective single transgenic mouse lines (i.e., BDNF^+/−^ and APP/PS1), regardless of the age of the animals. This finding is in line with other observations indicating that BDNF signaling is required for long-term but not short-term object memory (Seoane et al., 2011; Callaghan and Kelly, 2012). However, this speculation has to be assessed in future experimental approaches applying specific long-term and short-term memory tasks.

In conclusion, we could demonstrate that according to our hypothesis the chronic reduction of BDNF levels in our novel AD-mouse model indeed accelerated the onset of learning deficits in two way active avoidance learning. However, we could identify this acceleration only for TWAA learning but not for spatial learning in the MWM and for short-term memory in an object recognition task in the analyzed age groups. Overall, the creation of the triple transgenic mouse model was a further step to study the interrelation of BDNF and Aβ pathology *in vivo*. As the effects of chronic BDNF reduction vary between learning tasks requiring different complex brain networks this novel mouse model provides an interesting tool to further understand how differently regulated Aβ and BDNF expression relate to cognitive declines in AD pathology. Furthermore, this mouse model might be a promising model system to test BDNF-related treatment strategies against AD pathology. Thus, the APP/PS1-BDNF^+/−^-mouse model created in this study provides new opportunities to investigate the underlying molecular and cellular mechanisms of AD in more detail.

## Conflict of Interest Statement

The authors declare that the research was conducted in the absence of any commercial or financial relationships that could be construed as a potential conflict of interest.
